# Baseline Neurological Severity and Early Imaging Findings Predict 3-Month Functional Outcome in Anterior-Circulation Acute Ischemic Stroke: A Retrospective Cohort Study with One-Year Follow-Up

**DOI:** 10.3390/jcm15156016

**Published:** 2026-08-02

**Authors:** Abdullah Güzel, Elif Simin Issı, Sinem Yorgancı Ulaş, Usame Rakip, Ayşe Ertekin

**Affiliations:** 1 Department of Neurology, Faculty of Medicine, Afyonkarahisar Health Sciences University, Afyonkarahisar 03030, Türkiye; 2 Department of Neurosurgery, Faculty of Medicine, Afyonkarahisar Health Sciences University, Afyonkarahisar 03030, Türkiye; 3 Department of Emergency Medicine, Faculty of Medicine, Afyonkarahisar Health Sciences University, Afyonkarahisar 03030, Türkiye

**Keywords:** anterior circulation stroke, acute ischemic stroke, functional outcome, modified Rankin Scale, NIHSS, ASPECTS, prognostic model, prediction, stroke severity, TRIPOD

## Abstract

**Background:** Bedside prognostication after acute ischemic stroke remains challenging despite the availability of several composite scores, which often demand variables not routinely obtained at admission. Whether the combination of baseline neurological severity and early non-contrast computed tomography findings can support parsimonious, contemporaneous prediction of 3-month functional outcome is an open question, particularly given the well-documented overlap between clinical and imaging measures of stroke extent. **Methods:** We retrospectively analyzed 432 consecutive adults admitted with confirmed anterior-circulation acute ischemic stroke to a Turkish tertiary care university hospital between January 2022 and December 2025. The primary outcome was a poor functional outcome, defined as modified Rankin Scale (mRS) score 3–6 at 3 months. A prespecified multivariable logistic regression model incorporated three admission variables: baseline National Institutes of Health Stroke Scale (NIHSS) per five-point increment, Alberta Stroke Program Early CT Score (ASPECTS) per one-point increment, and age dichotomized at 65 years. Model performance was assessed with the area under the receiver operating characteristic curve (AUC), five-fold stratified cross-validation, and calibration testing. We additionally performed DeLong tests, net reclassification improvement (NRI), and integrated discrimination improvement (IDI) analyses to evaluate the incremental contribution of ASPECTS, and compared the parsimonious model against a comprehensive multivariable model containing ten predictors. **Results:** At 3 months, 252 patients (58.3%) had poor functional outcome, with 177 deaths (41.0%). Baseline NIHSS emerged as the dominant predictor (adjusted odds ratio [aOR] 4.16 per five-point increase, 95% confidence interval [CI] 2.79–6.22, *p* < 0.001), whereas ASPECTS retained no independent prognostic value after adjustment (aOR 0.99 per point, 95% CI 0.73–1.34, *p* = 0.952). Age ≥ 65 years was likewise non-significant (aOR 0.79, 95% CI 0.46–1.37, *p* = 0.405). The parsimonious model achieved excellent discrimination (AUC 0.904, 95% CI 0.876–0.932; cross-validated AUC 0.899, 95% CI 0.873–0.926) and good overall calibration on internal validation (calibration slope 0.958; calibration-in-the-large 0.009), although the Hosmer–Lemeshow test was significant (*p* = 0.001), reflecting localized miscalibration in the intermediate-risk range. Adding ASPECTS to a reduced model containing only NIHSS and age produced no meaningful discrimination gain (ΔAUC + 0.0003, DeLong *p* = 0.50; IDI ≈ 0), consistent with substantial variance sharing between NIHSS and ASPECTS (Spearman ρ = −0.89). A comprehensive model including infarct volume, Glasgow Coma Scale, reperfusion therapy, and comorbidities did not improve discrimination (AUC 0.905) and yielded a worse Akaike information criterion, supporting the sufficiency of the parsimonious model. **Conclusions:** In this Turkish tertiary center cohort, baseline NIHSS captured most of the prognostic information available at admission, and early ASPECTS added minimal independent value once neurological severity was accounted for. Findings should be interpreted in the context of the high case-mix severity of this referral population; external validation in independent, multicenter cohorts is required before clinical implementation is considered.

## 1. Introduction

Acute ischemic stroke continues to impose one of the largest disease burdens worldwide, with roughly 12 million new events and 7 million associated deaths reported annually [1,2]. Ischemic subtypes account for the majority of these cases and generate substantial downstream demand on rehabilitation, long-term care, and social welfare systems [3]. Even with the expansion of intravenous thrombolysis and endovascular thrombectomy over the last decade, a considerable proportion of survivors remain dependent at three months, and case-fatality rates in many real-world cohorts have plateaued rather than declined [4,5]. In this environment, reliable and rapidly obtainable prognostic information has become a practical necessity for treating clinicians, families weighing goals-of-care decisions, and health systems allocating post-acute resources.

The clinical value of accurate early prognostication is multifaceted. For patients and relatives, an informed estimate of the likelihood of functional independence enables shared decisions about ceiling of care, feeding, tracheostomy, and rehabilitation intensity [6]. For hospital services, risk stratification supports appropriate triage into stroke unit, intensive care, or palliative care pathways, and helps calibrate rehabilitation programs to expected trajectories [7]. For clinical trials, valid outcome prediction underpins eligibility criteria, stratified randomization, and adjusted analyses [8]. Prognostic tools that rely on variables already collected within the first hour of stroke care therefore have a clear place at the point of the bedside.

Among admission variables, the National Institutes of Health Stroke Scale (NIHSS) remains the reference measure of clinical stroke severity and has been repeatedly identified as the single strongest predictor of medium-term outcome across diverse populations and treatment eras [9,10]. The landmark Trial of Org 10172 in Acute Stroke Treatment (TOAST) reported a 17% reduction in the odds of excellent 3-month outcome for each additional NIHSS point [11], a gradient that has since been confirmed across varied clinical settings [10,12]. The persistence of this relationship across settings suggests that NIHSS reflects a physiologically integrated signal of tissue injury, brain reserve, and neurological compromise that is not fully captured by any single imaging biomarker.

Early non-contrast computed tomography quantified by the Alberta Stroke Program Early CT Score (ASPECTS) has emerged as the most widely used imaging index in hyperacute stroke care [13]. ASPECTS provides a semiquantitative measure of early ischemic change in the middle cerebral artery territory and has been embedded in patient selection criteria for endovascular therapy [14,15]. Its correlation with clinical severity, however, is high because larger infarct volumes typically generate more severe neurological deficits; several methodological reviews have documented that ASPECTS often loses independent prognostic significance once NIHSS is entered into multivariable models [16,17]. Whether ASPECTS provides genuinely additive information beyond NIHSS therefore remains contested and requires quantitative testing with contemporary methodology rather than simple regression *p*-values.

Numerous composite prognostic scores have been proposed to formalize outcome prediction, including the ASTRAL, DRAGON, iScore, and THRIVE scores [18,19,20,21]. These instruments perform respectably in validation studies but incorporate variables that are not universally available at admission, such as reperfusion status, occlusion location, or hyperdensity on baseline imaging [22,23]. External validation studies of ASTRAL and DRAGON have reported area under the curve (AUC) values in the range of 0.76–0.85 [24,25,26], while iScore and THRIVE typically achieve 0.72–0.83 [27]. These figures leave room for simpler, admission-only models that use variables reliably captured within minutes of arrival, particularly in health systems where advanced imaging or same-day thrombectomy is not universally accessible. A further consideration is that these instruments were derived predominantly in North American and European cohorts, and their external performance varies across settings and populations [8,22]. Multi-ethnic registry data underline the point: among 8825 South Asian patients in a Qatari stroke registry, ethnicity remained independently associated with poor 90-day outcome alongside admission NIHSS severity, and both case-fatality and functional recovery differed between ethnic groups [28]. Prognostic evidence generated in one center and one population therefore cannot be assumed to transport, and contemporary data from under-represented populations, including Türkiye, remain scarce.

The present study is positioned in this gap. Rather than proposing a novel composite score, we set out to quantify the relative and overlapping prognostic contributions of NIHSS, ASPECTS, and age in a contemporary Turkish tertiary center cohort, and to test explicitly whether ASPECTS offers incremental value beyond neurological severity when contemporary discrimination and reclassification metrics are applied. Four features distinguish this analysis from prior contributions. First, the cohort spans 2022–2025, covering the current era of routine mechanical thrombectomy and organized stroke networks in Türkiye, and thus reflects present-day case-mix rather than historical treatment landscapes. Second, we prespecified a parsimonious three-variable model on the basis of admission availability, and stress-tested it against a comprehensive ten-variable model to determine whether additional predictors materially change performance. Third, we applied formal nested-model comparison using DeLong testing, net reclassification improvement (NRI), integrated discrimination improvement (IDI), and variance inflation factors, moving beyond univariate *p*-values to characterize the ASPECTS–NIHSS relationship. Fourth, we report the analysis in alignment with the Transparent Reporting of a multivariable prediction model for Individual Prognosis Or Diagnosis (TRIPOD) framework in addition to the Strengthening the Reporting of Observational Studies in Epidemiology (STROBE) statement, providing the full prediction equation and a clinically usable risk table.

Our working hypothesis, informed by the mechanistic overlap between clinical severity and early ischemic change, was that NIHSS would dominate the model, ASPECTS would show attenuated independent effect, and a parsimonious admission-only equation would achieve discrimination comparable to more elaborate scores. The specific aims were, first, to develop and internally validate a three-variable prediction model for 3-month poor functional outcome; second, to quantify the incremental contribution of ASPECTS beyond NIHSS and age using DeLong, NRI, and IDI analyses; and third, to compare the parsimonious model against a comprehensive multivariable specification in terms of discrimination, calibration, and information criteria.

## 2. Materials and Methods

### 2.1. Study Design and Setting

This retrospective cohort study was conducted at Afyonkarahisar Health Sciences University Hospital, a tertiary care university hospital with a comprehensive stroke center, between January 2022 and December 2025. The study protocol was approved by the Institutional Ethics Committee (Afyonkarahisar Health Sciences University Non-Interventional Scientific Research Ethics Committee, Decision No: 367, Meeting No: 2026/9, Date: 8 May 2026), and the requirement for informed consent was waived due to the retrospective nature of the study. This study was reported in accordance with the Strengthening the Reporting of Observational Studies in Epidemiology (STROBE) guidelines [29] (Supplementary Table S1).

### 2.2. Patient Selection

**Figure 1:**
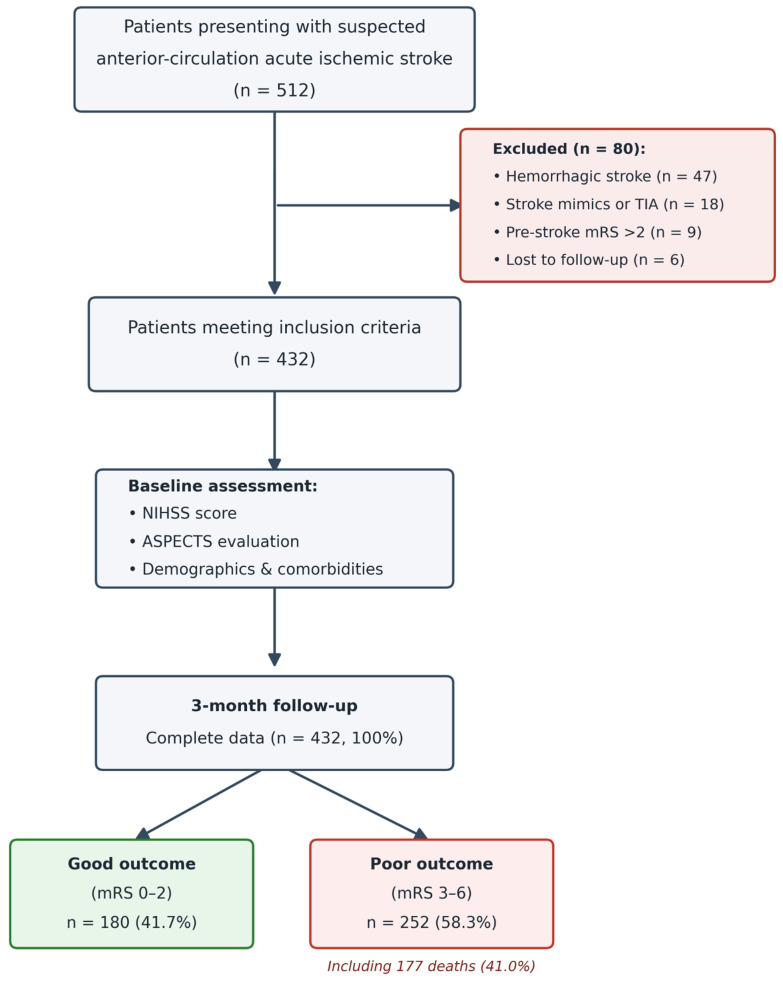
Patient flow diagram. Of 512 patients screened for suspected anterior-circulation acute ischemic stroke, 432 met the inclusion criteria after exclusions.

We consecutively enrolled adult patients (≥18 years) presenting to the emergency department with acute ischemic stroke confirmed by neuroimaging. The inclusion criteria were: (1) acute ischemic stroke in the anterior (carotid) circulation diagnosed within 24 h of symptom onset, (2) baseline NIHSS assessment performed at admission, (3) non-contrast computed tomography (CT) performed at admission with ASPECTS evaluation, and (4) available 3-month follow-up data. The screening frame was restricted a priori to middle cerebral artery (MCA) territory ischemic stroke, because the standard Alberta Stroke Program Early CT Score was developed for early ischemic change in the MCA territory. Accordingly, isolated anterior cerebral artery infarctions and posterior-circulation (vertebrobasilar) events were outside the sampling frame; a small number of patients with combined MCA and anterior cerebral artery involvement were retained, as ASPECTS remains applicable to the MCA component. These out-of-frame presentations are not counted among the exclusions shown in Figure 1. Exclusion criteria included: (1) hemorrhagic stroke, (2) stroke mimics or transient ischemic attack (grouped together, as neither produces a completed ischemic infarct scorable by ASPECTS), (3) significant pre-stroke disability (mRS > 2) [30], (4) missing baseline neurological assessment, and (5) being lost to follow-up before 3 months.

### 2.3. Data Collection

Demographic data, medical history, and clinical variables were extracted from electronic medical records by trained investigators. Baseline neurological severity was assessed using the NIHSS by certified stroke neurologists at emergency department presentation. The Glasgow Coma Scale (GCS) was recorded as part of routine assessment but was not included in the primary model due to collinearity with NIHSS. All patients underwent non-contrast CT at admission. Baseline ASPECTS was evaluated on the admission non-contrast CT by three stroke neurologists who were blinded to 3-month functional outcome, with the final score assigned by consensus. Because the score was assigned by consensus rather than by independent readings, an inter-rater agreement statistic was not computed. Infarct volume was calculated using the ABC/2 method on follow-up imaging [31]. Stroke etiology was classified according to the Trial of Org 10172 in Acute Stroke Treatment (TOAST) criteria [32]. The three primary predictors (baseline NIHSS, ASPECTS, and age) and the primary outcome were complete for all 432 included patients, with no missing values; multiple imputation was therefore not required.

### 2.4. Outcomes

The primary outcome was poor functional outcome at 3 months, defined as modified Rankin Scale (mRS) score 3–6. Good functional outcome was defined as mRS 0–2, indicating functional independence. All percentages for functional outcomes are calculated over the entire cohort of 432 patients as the denominator. Secondary outcomes included 3-month mortality, 1-year functional outcome, and functional trajectory over time. Functional outcomes were assessed by certified stroke nurses or physicians during outpatient visits or structured telephone interviews at discharge, 30 days, 3 months, 6 months, and 1 year after stroke onset. Because outcome data were collected as part of routine clinical care and abstracted retrospectively, it could not be verified whether outcome assessors were blinded to baseline NIHSS and ASPECTS values; this is acknowledged as a limitation. Exact discharge and 30-day mRS values could not be retrieved for 120 patients who were discharged alive but died between 30 days and 3 months; their mRS band at those two timepoints was assigned using a post hoc, rule-based reconstruction described below in Section 3.8. The 6-month mRS was recorded for all patients; however, a dedicated 6-month date-of-death field was not available, so the exact timing of the 14 deaths between 3 months and 1 year could not be verified. The recorded 6-month mRS indicates these 14 patients were alive at 6 months, and their deaths are therefore shown in the 1-year row.

### 2.5. Statistical Analysis

Continuous variables were summarized as mean ± standard deviation (SD) to facilitate comparison with prior stroke cohorts, which conventionally report these measures; normality was evaluated using the Shapiro–Wilk test, and because several variables (including NIHSS, ASPECTS, and infarct volume) were non-normally distributed, all between-group comparisons of continuous variables used the non-parametric Mann–Whitney U test regardless of distribution. Categorical variables were summarized as frequencies and percentages. Between-group comparisons used the Mann–Whitney U test for continuous variables and the chi-square or Fisher’s exact test for categorical variables, as appropriate. A two-sided *p*-value < 0.05 was adopted as the threshold for statistical significance throughout.

Univariate logistic regression screened candidate admission variables for association with 3-month poor functional outcome. For the primary multivariable model, three variables were prespecified on the basis of routine admission availability and prior evidence: baseline NIHSS (entered per 5-point increase to provide a clinically meaningful odds ratio), ASPECTS (per 1-point increase), and age dichotomized at 65 years, a conventional threshold in the stroke prognostic literature. This parsimonious specification was selected a priori to minimize overfitting, given the anticipated event rate, and to maximize applicability at the bedside.

Model discrimination was quantified using the area under the receiver operating characteristic curve (AUC) with a 95% confidence interval (CI) derived from 1000 bootstrap replications. Internal validation used 5-fold stratified cross-validation, with fold-level AUCs reported alongside the mean cross-validated AUC. Bootstrap optimism correction was additionally performed to estimate the shrinkage of apparent model performance [33]. Model calibration was evaluated using the Hosmer–Lemeshow goodness-of-fit test, calibration slope, calibration-in-the-large, and visual inspection of a calibration plot (Supplementary Figure S1). The optimal probability threshold for classifying poor outcome was selected using the Youden index, and sensitivity, specificity, positive predictive value, and negative predictive value at this threshold were derived.

To rigorously test whether ASPECTS contributes independent prognostic information beyond NIHSS and age, we performed formal nested-model comparisons. Two nested models were fitted: a reduced model containing NIHSS (per 5-point increment) and age (dichotomized at 65 years), and a full model additionally incorporating ASPECTS. The DeLong test [34] was applied to compare AUCs between nested models. The category-free (continuous) net reclassification improvement (NRI) was computed as the sum of events reclassified toward higher risk and non-events reclassified toward lower risk. Integrated discrimination improvement (IDI) was calculated as the difference in the mean predicted probabilities of the two models, separately for events and non-events [35]. Variance inflation factors (VIFs) and Spearman rank correlations among NIHSS, ASPECTS, GCS, and infarct volume were computed to characterize multicollinearity and shared variance among clinical and imaging measures of stroke extent.

To evaluate whether the parsimonious model discarded meaningful prognostic information, a comprehensive multivariable logistic regression was constructed containing the three primary predictors plus infarct volume (as a descriptive baseline variable acknowledged to derive from follow-up imaging), GCS, hypertension, diabetes mellitus, atrial fibrillation, intravenous thrombolysis, and mechanical thrombectomy. Discrimination (AUC), calibration, and model fit statistics (Akaike information criterion; AIC) were compared between the parsimonious and comprehensive models. TOAST etiology was examined descriptively rather than as a model term to avoid instability from small subgroup counts.

Because the dichotomization of continuous variables can attenuate estimates and mask nonlinear relationships, we conducted sensitivity analyses in which age was alternately modeled as (i) a dichotomous variable at 65 years (primary specification), (ii) a continuous linear term, and (iii) a polynomial term (linear plus quadratic). AUC and AIC were compared across specifications.

To align with TRIPOD reporting expectations [36], the full logistic regression equation, including the intercept and coefficient of each predictor, is provided. A bedside risk table cross-tabulating predicted probability of poor outcome by NIHSS category, ASPECTS category, and age group is presented in Supplementary Table S7 to facilitate clinical use. All analyses were performed in Python 3.10 (Python Software Foundation, Wilmington, DE, USA) using scikit-learn (version 1.2), statsmodels (version 0.14), scipy (version 1.10), and lifelines (version 0.27). Bootstrap and cross-validation procedures used a fixed random seed (42) for reproducibility. The 95% confidence interval for the cross-validated AUC was obtained from the 2.5th and 97.5th percentiles of 1000 bootstrap resamples of the out-of-fold predictions. Because all required components were available, the THRIVE score was calculated for each patient according to its original definition using age, baseline NIHSS, hypertension, diabetes mellitus, and atrial fibrillation. Its discrimination for 3-month poor functional outcome was assessed using the AUC with a 95% bootstrap confidence interval and compared with that of the prespecified three-variable model using DeLong’s test for correlated ROC curves.

### 2.6. Sample Size Consideration

Under the conventional rule of at least 10 outcome events per predictor parameter, the three-predictor primary model required approximately 30 poor-outcome events. With 252 poor outcomes, our cohort substantially exceeded this threshold.

## 3. Results

### 3.1. Study Population

During the study period, 512 patients with suspected anterior-circulation acute ischemic stroke were screened. After excluding 47 patients with hemorrhagic stroke, 18 with stroke mimics, nine with significant pre-stroke disability, and six lost to follow-up, 432 patients were included in the final analysis (Figure 1). Complete 3-month follow-up data were available for all included patients (100%).

### 3.2. Baseline Characteristics

Baseline demographic, clinical, and imaging characteristics of the cohort, stratified by 3-month functional outcome, are summarized in Table 1. The mean age was 65.1 ± 12.9 years, and 245 patients (56.7%) were male. The median time from symptom onset to hospital arrival was 3.74 h (IQR: 1.49–6.82). The most common comorbidities were hypertension (71.8%), atrial fibrillation (38.0%), and diabetes mellitus (36.3%). At presentation, the mean NIHSS score was 12.7 ± 11.1, and the mean ASPECTS was 7.2 ± 2.7. Regarding NIHSS severity categories, 130 patients (30.1%) had minor stroke (NIHSS 0–4), 167 (38.7%) had moderate stroke (NIHSS 5–15), 65 (15.0%) had moderate-severe stroke (NIHSS 16–24), and 70 (16.2%) had severe stroke (NIHSS > 24). Sixty-eight patients (15.7%) received intravenous thrombolysis, and 53 (12.3%) underwent mechanical thrombectomy. Stroke etiology according to TOAST classification was cryptogenic in 130 patients (30.1%), cardioembolic in 105 (24.3%), large artery atherosclerosis in 79 (18.3%), lacunar in 70 (16.2%), and other determined etiology in 48 (11.1%).

### 3.3. Primary Outcome and Group Comparison

At 3-month follow-up, 180 patients (41.7%) achieved good functional outcome (mRS 0–2) while 252 (58.3%) had poor functional outcome (mRS 3–6), including 177 deaths (mRS 6, 41.0%). The full 3-month mRS distribution was: mRS 0, 76 patients (17.6%); mRS 1, 63 (14.6%); mRS 2, 41 (9.5%); mRS 3, 32 (7.4%); mRS 4, 29 (6.7%); mRS 5, 14 (3.2%); and mRS 6, 177 (41.0%).

Patients who developed poor outcome were older (66.9 ± 13.2 versus 62.7 ± 12.1 years, *p* = 0.001) and more likely to be aged ≥65 years (56.3% versus 45.0%, *p* = 0.026). They also had substantially higher NIHSS (18.4 ± 10.9 versus 4.6 ± 4.1, *p* < 0.001), lower GCS (9.8 ± 3.4 versus 13.2 ± 1.8, *p* < 0.001), lower ASPECTS (6.0 ± 2.8 versus 9.0 ± 1.0, *p* < 0.001), and larger follow-up infarct volumes (98.7 ± 80.4 versus 28.7 ± 23.6 mL, *p* < 0.001) (Table 1). Prevalences of hypertension (*p* = 0.427), diabetes mellitus (*p* = 0.554), and previous stroke (*p* = 0.631) did not differ significantly between outcome groups. Reperfusion therapy was more frequently administered to patients who ultimately had poor outcome (intravenous alteplase, 19.8% versus 10.0%, *p* = 0.008; mechanical thrombectomy, 17.5% versus 5.0%, *p* < 0.001), a pattern attributable to confounding by indication because more severe strokes are prioritized for reperfusion.

### 3.4. Outcomes Stratified by NIHSS Category

**Figure 2:**
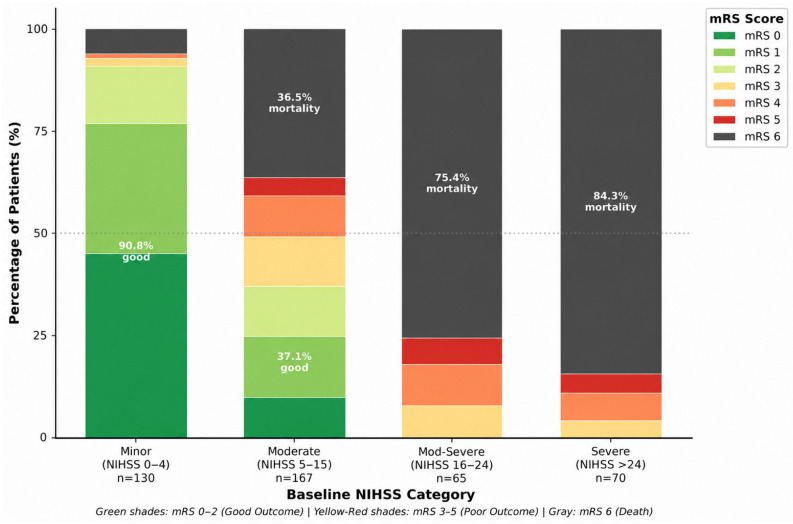
Distribution of 3-month modified Rankin Scale (mRS) scores by baseline NIHSS category. The stacked bar chart demonstrates a clear dose–response relationship between initial stroke severity and 3-month functional outcome.

A monotonic dose–response relationship was observed between the admission NIHSS category and the 3-month outcome (Table 2, Figure 2). Among minor strokes (NIHSS 0–4), 90.8% achieved good outcome with only 6.2% dying by 3 months. In contrast, no patient with moderate-to-severe (NIHSS 16–24) or severe stroke (NIHSS > 24) achieved functional independence at 3 months, with respective mortality rates of 75.4% and 84.3%. Patients with moderate stroke (NIHSS 5–15) displayed intermediate results, with 37.1% attaining good outcome and 36.5% dying within 3 months.

### 3.5. Outcomes Stratified by ASPECTS Category

A clinically meaningful association between admission ASPECTS category and 3-month functional outcome was also observed (Table 3). Among patients with ASPECTS 8–10, 64.8% achieved good outcome and 20.0% died within 3 months. No patient with ASPECTS ≤ 5 attained functional independence, and 3-month mortality in this group reached 81.4%. Patients with ASPECTS 6–7 showed intermediate results, with 18.8% good outcome and 59.4% mortality.

### 3.6. Multivariable Analysis

In the primary multivariable logistic regression model, baseline NIHSS was the dominant independent predictor of poor 3-month functional outcome (adjusted odds ratio [aOR] 4.16 per 5-point increase, 95% CI 2.79–6.22, *p* < 0.001) (Table 4). ASPECTS lost independent prognostic significance after adjustment for NIHSS and age (aOR 0.99 per point, 95% CI 0.73–1.34, *p* = 0.952). Age ≥ 65 years likewise did not demonstrate an independent association (aOR 0.79, 95% CI 0.46–1.37, *p* = 0.405).

### 3.7. Model Performance and Calibration

**Figure 3:**
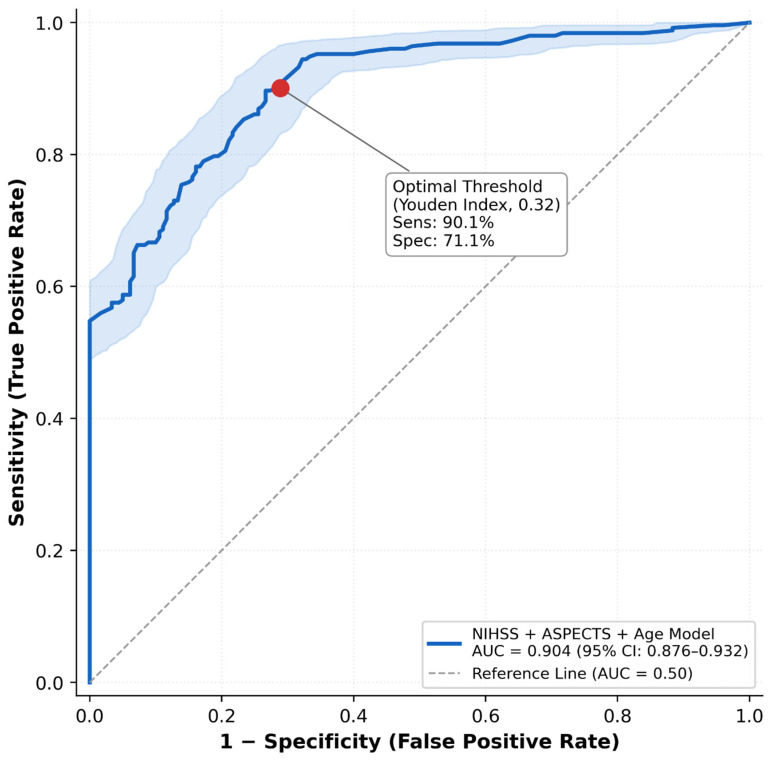
Receiver operating characteristic (ROC) curve for the prediction model. The model including baseline NIHSS (per 5-point increase), ASPECTS (per 1-point increase), and age ≥ 65 years demonstrated excellent discrimination (AUC 0.904, 95% CI 0.876–0.932).

**Figure 4:**
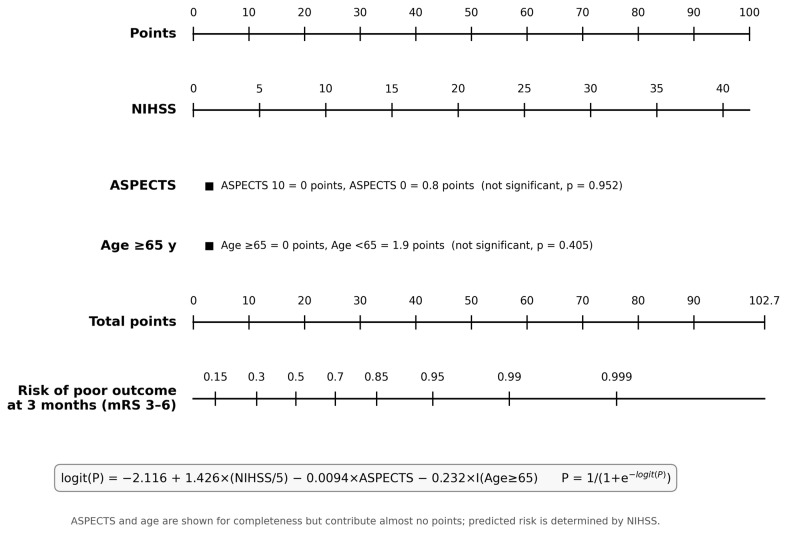
Nomogram for predicting poor functional outcome (mRS 3–6) at 3 months. For each predictor, points are read from the uppermost axis, summed to give the total points, and converted to a predicted probability on the lowest axis. The nomogram is derived from the fitted multivariable model reported in Table 4 (n = 432). ASPECTS and age ≥ 65 years are displayed for completeness but were not statistically significant; across their entire range they shift the total by fewer than 3 points, so predicted risk is determined almost entirely by baseline NIHSS. The nomogram is intended as an aid to risk stratification—that is, to ranking patients from lower to higher risk—rather than as a source of exact individual probability estimates. Calibration was close to ideal in the low- and high-risk ranges but deviated in the intermediate-risk range (Supplementary Figure S1), so values read from the middle of the risk axis carry greater uncertainty than their apparent precision suggests. The corresponding bedside risk table is provided in Supplementary Table S7. NIHSS, National Institutes of Health Stroke Scale; ASPECTS, Alberta Stroke Program Early CT Score; mRS, modified Rankin Scale.

The prediction model achieved excellent discrimination in the derivation cohort (AUC 0.904, 95% CI 0.876–0.932; Figure 3). Five-fold stratified cross-validation confirmed model stability (mean cross-validated AUC 0.899, 95% CI 0.873–0.926; individual fold AUCs 0.884–0.924), and bootstrap optimism correction yielded a shrinkage-adjusted AUC of 0.900, indicating minimal overfitting. Using the Youden-derived probability threshold of 0.32, the model demonstrated 90.1% sensitivity and 71.1% specificity, with corresponding positive predictive value of 81.4% and negative predictive value of 83.7%. Calibration was assessed using internally validated (5-fold cross-validated) measures, because apparent calibration statistics computed in the derivation sample are uninformative by construction (slope exactly 1.00, calibration-in-the-large exactly 0.00). The internally validated calibration slope was 0.958 and calibration-in-the-large was 0.009, indicating close overall agreement between observed and predicted probabilities. The Hosmer–Lemeshow test was, however, statistically significant (χ^2^ = 25.8, df = 8, *p* = 0.001), reflecting localized deviation in the intermediate-risk range, where the model over-predicted observed risk in the third decile of predicted risk (five events observed versus 9.8 expected) and under-predicted it in the fourth (23 versus 14.4 expected); discrimination and overall calibration were otherwise preserved. The flexible calibration curve is provided in Supplementary Figure S1. The full prediction equation is: logit(P) = −2.116 + 1.426 × (NIHSS/5) − 0.0094 × ASPECTS − 0.232 × I(Age ≥ 65 years), with P(poor outcome at 3 months) = 1/(1 + exp(−logit(P))); A nomogram derived from this model is presented in Figure 4, and a corresponding bedside risk table stratified by NIHSS category, ASPECTS category, and age group is provided in Supplementary Table S7. A complete TRIPOD checklist is provided in Supplementary Table S8.

### 3.8. Functional Trajectory

**Figure 5:**
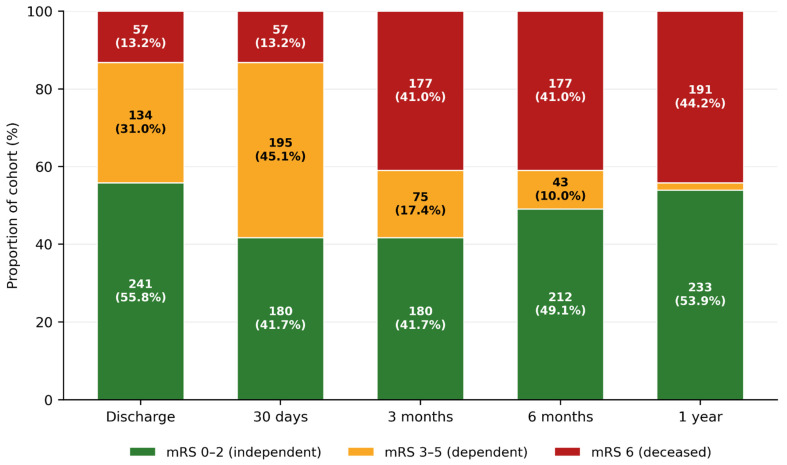
Functional status across the 12-month follow-up window. Stacked bars show the number and proportion of the analytic cohort (n = 432) within each modified Rankin Scale (mRS) band at each timepoint: mRS 0–2 (functionally independent), mRS 3–5 (dependent), and mRS 6 (deceased). Exact discharge and 30-day mRS values were unavailable for 120 patients who died between 30 days and 3 months; their placement in the mRS 3–5 band at those timepoints reflects the post hoc, rule-based reconstruction described in the Table 5 footnote and should not be interpreted as an independently observed transition. Three-month, 6-month, and 1-year mRS values were recorded for all patients. The number of patients with mRS 0–2 increased from 180 at 3 months to 212 at 6 months and 233 at 1 year, consistent with functional improvement among survivors. Between 6 months and 1 year, 24 patients improved and 3 deteriorated, producing a net increase of 21 patients with mRS 0–2. The 6-month mRS indicated that the 14 patients who died between 3 months and 1 year were alive at the 6-month assessment; exact dates of death were unavailable. No patient moved from mRS 6 to a lower category at any subsequent timepoint. mRS, modified Rankin Scale.

The functional trajectory of the cohort across the 12-month follow-up window is summarized in Table 5 and Figure 5. Functional status is reported as the number of patients within each mRS band (0–2, 3–5, 6) rather than as a mean mRS score, because mRS 6 denotes death and a mean computed across survivors and decedents conflates functional recovery with the accumulation of mortality. In-hospital mortality was 57 (13.2%), established from the recorded discharge destination and concordant with the 30-day mortality field in 57 of 57 cases. At discharge, 241 patients (55.8%) had good functional outcome (mRS 0–2), 134 (31.0%) were dependent (mRS 3–5), and 57 (13.2%) had died. By 30 days, good outcome had declined to 180 (41.7%) with no further deaths, as 63 patients deteriorated from independence to dependence and two improved. All 241 patients recorded as independent at discharge were discharged home, and none were transferred to a nursing facility or to palliative care, indicating that the discharge mRS assignment was internally consistent with the discharge destination. The 63 patients who subsequently deteriorated differed systematically from the 178 who remained independent: they had sustained more severe index strokes (baseline NIHSS 11.8 vs. 4.5, *p* < 0.001; ASPECTS 7.4 vs. 9.0, *p* < 0.001; infarct volume 64.8 vs. 28.4 mL, *p* < 0.001), were far more likely to have undergone reperfusion therapy (mechanical thrombectomy 28.6% vs. 5.1%, *p* < 0.001; intravenous thrombolysis 23.8% vs. 9.6%, *p* = 0.008), had longer hospital stays (11.6 vs. 8.8 days, *p* < 0.001), and were discharged at the margin of independence rather than in full recovery (discharge NIHSS 4.7 vs. 1.5, *p* < 0.001; discharge Barthel Index 91.9 vs. 95.1, *p* = 0.005). They also experienced more in-hospital pneumonia (23.8% vs. 10.7%, *p* = 0.018). Vascular risk factors did not differ between the two groups (all *p* > 0.15). Among the patients with observed discharge and 30-day mRS, this pattern was consistent with early functional decline in a subgroup with more severe strokes, larger infarct volumes, higher reperfusion-treatment rates, and greater residual deficit at discharge who achieved marked early neurological improvement and were discharged home while still close to the dependence threshold, rather than an artifact of optimistic mRS scoring at discharge. Between 30 days and 3 months, 120 further deaths occurred; because these patients were classified into the mRS 3–5 band at 30 days precisely on the basis of their subsequent death (their exact 30-day mRS being unavailable), their location in that band is in part a consequence of the reconstruction rule rather than an independent observation. Of these 120 patients, 98 (81.7%) had been discharged to palliative care or a nursing facility, and 22 to home. Good outcome subsequently recovered to 212 (49.1%) at 6 months through improvement among 32 survivors, and to 233 (53.9%) at 1 year (24 patients improved and 3 deteriorated, a net increase of 21), while cumulative all-cause mortality reached 191 (44.2%) following 14 late deaths between 3 months and 1 year. No patient transitioned from mRS 6 to a lower score at any timepoint.

### 3.9. Incremental Value of ASPECTS Beyond NIHSS

To formally evaluate the added prognostic contribution of ASPECTS, we compared nested models. A reduced model containing NIHSS and age achieved an AUC of 0.903, while the full model additionally incorporating ASPECTS achieved 0.904 (ΔAUC + 0.0003; DeLong z = 0.68, *p* = 0.497) (Supplementary Table S2). A likelihood ratio test comparing the same nested models was likewise non-significant (χ^2^ = 0.004, df = 1, *p* = 0.952), indicating that the addition of ASPECTS did not improve model fit. The category-free NRI was 0.225 (95% CI 0.04–0.44), driven by modest event reclassification (+10.3%) and non-event reclassification (+12.2%). The IDI was negligible (approximately zero), indicating no measurable improvement in the separation of predicted probabilities between events and non-events (Supplementary Table S3). Multicollinearity assessment demonstrated moderate-to-substantial shared variance among clinical and imaging measures of stroke extent: Spearman rank correlations were −0.89 between NIHSS and ASPECTS, −0.78 between NIHSS and GCS, and −0.84 between ASPECTS and infarct volume (Supplementary Table S4). Variance inflation factors were 6.0 for NIHSS and 6.0 for ASPECTS in the primary model. Together, these results indicate that the apparent non-significance of ASPECTS in the multivariable model reflects shared variance with NIHSS rather than absence of prognostic content, supporting an interpretation of biological redundancy rather than clinical irrelevance.

### 3.10. Comparison with Comprehensive Model

To determine whether the parsimonious model discarded meaningful prognostic information, a comprehensive multivariable model was fitted incorporating the three primary predictors plus infarct volume, GCS, hypertension, diabetes mellitus, atrial fibrillation, intravenous thrombolysis, and mechanical thrombectomy. The comprehensive model achieved an AUC of 0.905, essentially identical to the parsimonious model (ΔAUC + 0.0016), and had a higher AIC (350.8 versus 341.6), indicating inferior model fit once complexity was penalized (Supplementary Table S5). Cross-validated AUC of the comprehensive model was 0.896 ± 0.017, and its cross-validated calibration was slightly inferior to that of the parsimonious model (calibration slope 0.887 versus 0.958; Brier score 0.134 versus 0.130; Supplementary Table S5). For reference, the NIHSS-only model had the lowest AIC (338.3); the three-predictor specification was retained as the prespecified study model because the primary objective was to quantify the incremental contribution of ASPECTS and age beyond NIHSS, which requires those terms to be present. These findings support the sufficiency of the parsimonious admission-based model for 3-month functional outcome prediction in this cohort.

### 3.11. Age Modeling Sensitivity Analysis

To address the concern that dichotomizing age at 65 years may distort estimated effects, we compared three age specifications (Supplementary Table S6). Dichotomous age (≥65 years) yielded AUC 0.904 and AIC 341.6; continuous linear age yielded AUC 0.903 and AIC 341.5; and a polynomial specification (age plus age^2^) yielded AUC 0.903 and AIC 343.5. As a continuous variable, age carried a per-year adjusted OR of 0.99 (95% CI 0.97–1.01, *p* = 0.37), which did not reach statistical significance. These sensitivity analyses indicate that the null independent age association observed in the primary model is not an artifact of the chosen dichotomization threshold.

### 3.12. Comparison with the THRIVE Score

The THRIVE score was calculable for all 432 patients. It achieved an AUC of 0.792 (95% CI 0.751–0.834) for predicting 3-month poor functional outcome. The prespecified NIHSS–ASPECTS–age model demonstrated higher discrimination (AUC 0.904), and the difference between the correlated ROC curves was statistically significant (DeLong *p* < 0.001). A summary of this comparison is provided in Supplementary Table S10.

### 3.13. Exploratory 12-Month Outcome Model

Because complete 12-month follow-up was available, we additionally fitted the same three-variable admission model to poor functional outcome (mRS 3–6) at 1 year as an exploratory analysis (Supplementary Table S9). The model was estimable (199 events versus 233 non-events) and discriminated adequately (AUC 0.868), although less well than at 3 months (AUC 0.904), and the effect of baseline NIHSS attenuated over time (aOR 2.97 per 5-point increase, 95% CI 2.16–4.09, *p* < 0.001, versus 4.16 at 3 months). ASPECTS and age ≥ 65 years remained non-significant. This analysis is reported as exploratory rather than as a second validated prediction model, for a reason that is intrinsic to the outcome structure at 1 year rather than to the modeling approach. Of the 199 patients with poor outcome at 12 months, 191 (96.0%) had died, leaving only eight surviving patients in the mRS 3–5 stratum. A model for poor 12-month functional outcome in this cohort is therefore, in substance, a model of 12-month mortality: predicted probabilities from the 12-month poor-outcome model and from a 12-month all-cause mortality model fitted to the same predictors were almost perfectly correlated (Pearson r = 0.999), and within the poor-outcome group the model could not distinguish the 8 surviving dependent patients from the 191 decedents (AUC 0.563, approximately chance). The 12-month data reported here therefore describe the observed recovery trajectory of this cohort and support the conclusion that early prediction of 3-month outcome should not be read as a fixed long-term prognosis; they do not constitute a validated instrument for predicting 12-month functional recovery, which would require a cohort containing a substantially larger number of surviving dependent patients.

## 4. Discussion

In this contemporary Turkish tertiary center cohort of 432 consecutive adults with anterior-circulation acute ischemic stroke, baseline NIHSS emerged as the dominant admission predictor of 3-month poor functional outcome, while early ASPECTS added no measurable independent value once neurological severity was accounted for. A prespecified three-variable admission model achieved excellent internal discrimination (AUC 0.904, cross-validated 0.899), remained well-calibrated overall on internal validation (calibration slope 0.958; calibration-in-the-large 0.009), with localized miscalibration confined to the intermediate-risk range (Hosmer–Lemeshow *p* = 0.001), and was internally validated with minimal overfitting (bootstrap optimism 0.004).

Because the individual finding that NIHSS predicts outcome is well-established, several elements of the present analysis warrant explicit statement of what is genuinely new. First, the cohort covers 2022–2025 and thus reflects the current era of routine mechanical thrombectomy and organized stroke networks in a Turkish tertiary setting; contemporaneous data from this population are scarce in the international literature. Second, we quantified the incremental prognostic contribution of ASPECTS beyond NIHSS using formal nested-model comparisons—DeLong testing, category-free NRI, and IDI—rather than relying only on the *p*-value of the ASPECTS coefficient in a single multivariable model. Third, we stress-tested the parsimonious specification against a comprehensive ten-variable model to demonstrate that adding infarct volume, GCS, comorbidities, and reperfusion therapy does not improve either discrimination or information criteria in this cohort. Fourth, we align reporting with the TRIPOD statement and provide the full logistic equation and a bedside risk table, enabling readers to apply the model at the point of care and future investigators to validate it externally. These elements move the analysis beyond a repetition of the NIHSS–outcome relationship into a quantitative characterization of how much prognostic content NIHSS actually carries relative to imaging and demographic anchors in a current stroke-care environment.

### 4.1. Contextualizing the High Case-Mix Severity

Before interpreting these findings, the specific contribution of this study should be stated explicitly, because the dominance of baseline NIHSS as a prognostic marker itself is long established. The contribution here is not the rediscovery of that association but three other elements. First, this is a contemporary consecutive single-center cohort from Türkiye, a population under-represented in the prognostic stroke literature, characterized in sufficient detail for its case-mix to be compared with that of other settings. Second, rather than assuming that early CT adds prognostic information, the study quantifies the incremental value of ASPECTS over NIHSS directly, using four complementary analytical approaches—nested-model discrimination (DeLong), likelihood ratio testing, reclassification (NRI and IDI), and formal collinearity assessment—which, together, distinguish biological redundancy from clinical irrelevance; this quantification, rather than the qualitative observation, is what the existing literature lacks. Third, the resulting model is deliberately parsimonious and fully reported, with the complete equation, intercept, nomogram, and bedside risk table provided, so that it can be reproduced and tested by others rather than merely cited. We do not claim that the model is ready for local clinical deployment: as set out below, calibration was imperfect in the intermediate-risk range and validation was internal only. A 3-month all-cause mortality of 41.0% in this cohort is substantially higher than that reported in many population-based ischemic stroke cohorts. Crude mortality figures are therefore of limited value as stand-alone indicators of care quality unless case-mix is explicitly accounted for [37]. Several features of the local care pathway plausibly contribute to this observation and should be considered when interpreting model performance. First, Afyonkarahisar Health Sciences University Hospital serves as a referral center for a wide geographic catchment area and may therefore receive a disproportionate share of severe strokes transferred from surrounding secondary hospitals; although transfer and large-vessel-occlusion rates were not systematically recorded, this referral pattern may have contributed to a case-mix enriched for severe presentations. Second, the median symptom-onset-to-hospital-arrival time in this cohort was 3.74 h (IQR 1.49–6.82), and the upper quartile exceeded 6 h, suggesting appreciable presentation delay in a subset of patients, particularly those transferred from peripheral facilities. Third, in the regional practice pattern milder strokes without persistent deficits may be discharged directly from the emergency department without stroke-unit admission, which could further shift the analytic cohort toward greater severity; this pattern was not quantified in the present dataset. Fourth, patients with NIHSS 16–24 and NIHSS > 24 collectively accounted for 31.2% of the analytic sample, a proportion higher than in most published unselected stroke registries. These case-mix features have two implications for the interpretation of our results. First, the AUC of 0.904 is likely to be enhanced by the wide separation between minor and severe strokes in our sample. Discrimination is not a property of a model alone: the c-statistic depends on the heterogeneity of the case-mix in which it is measured as well as on the correctness of the regression coefficients, so a model applied to a population with a wider spread of predicted risk will yield a higher c-statistic even when its coefficients are unchanged [38]. Accordingly, models developed in populations with a more balanced severity distribution may perform less well, and the AUC values reported here should not be interpreted as directly transportable to community-based cohorts. For the same reason, the magnitude of our AUC should not be read as evidence of superiority over models reported in other cohorts, whose case-mix differs. Second, the striking absence of functional independence at 3 months among patients with NIHSS ≥ 16 in our sample is consistent with the combined effect of severe initial injury, referral-related delays, and limited access to hyperacute reperfusion for a subset of transferred patients; this observation should be understood as reflecting the local care pathway rather than an intrinsic ceiling for severe stroke recovery.

### 4.2. Interpreting the Limited Independent Value of ASPECTS

Our formal nested-model analysis demonstrated that adding ASPECTS to a model containing NIHSS and age produced a change in AUC of only +0.0003 (DeLong *p* = 0.497) and an IDI approximately equal to zero. Spearman rank correlation between NIHSS and ASPECTS was −0.89, and the variance inflation factor of ASPECTS in the primary model was 6.0, indicating moderate-to-substantial multicollinearity. Thus, ASPECTS did not improve the AUC, the likelihood-based model fit, or the IDI, despite a positive category-free NRI whose clinical significance is uncertain given the offsetting DeLong, likelihood ratio, and IDI results. These findings support the interpretation that ASPECTS and NIHSS capture largely overlapping information about the extent of ischemic injury, with NIHSS integrating this information into a clinically graded severity score. Similar attenuation of the independent effect of ASPECTS after adjustment for clinical severity has been reported in the methodological reviews of Schröder and Thomalla and of Soliman and colleagues [14,16].

We emphasize that a non-significant coefficient in a multivariable model does not imply that ASPECTS lacks clinical value. In hyperacute settings where mechanical thrombectomy decisions must be made in minutes, ASPECTS remains clinically relevant for imaging-based treatment selection and characterization of early ischemic extent. The finding presented here is narrower: for the specific task of predicting 3-month functional outcome from admission variables in this consecutive single-center cohort, ASPECTS does not carry information that is additional to NIHSS. This distinction is important because prognostic and selection tasks impose different informational requirements, and imaging findings that are indispensable for one may be redundant for the other.

### 4.3. Comparison with Existing Prognostic Scores

Multiple composite scores have been developed and externally validated for stroke outcome prediction, including ASTRAL (validation AUCs 0.76–0.85), DRAGON (0.80–0.84), iScore (0.72–0.83), and THRIVE (0.72–0.83) [18,19,20,21,22,23,24,25,26,27]. Simplified instruments combining only age and neurological severity, such as SPAN-100, follow a rationale closely comparable to the present specification [39]. Systematic appraisals of the stroke prognostic modeling literature have nevertheless highlighted heterogeneous methodology, frequent absence of internal validation, and incomplete reporting as recurrent shortcomings [40,41]. The AUC observed in the present cohort (0.904) falls within or above the ranges reported in those studies, but the values are not directly comparable because patient populations, outcome definitions, follow-up windows, and treatment eras differ substantially. Rather than claiming superiority, we position the present model as demonstrating that a parsimonious admission-only specification can achieve discrimination within or above the range reported for established composite scores in a contemporary Turkish tertiary setting. Among these instruments, the THRIVE score could be computed in our dataset because all of its components (age, baseline NIHSS, hypertension, diabetes mellitus, and atrial fibrillation) were available. THRIVE discriminated 3-month poor functional outcome with an AUC of 0.792 (95% CI 0.751–0.834), which was lower than that of the prespecified model (AUC 0.904; DeLong *p* < 0.001). This difference may primarily reflect the finer-grained use of NIHSS and differences in predictor coding and model specification; it should not be attributed specifically to ASPECTS, because ASPECTS did not improve discrimination in the nested-model comparison. Full head-to-head comparison against ASTRAL, DRAGON, and iScore was not possible because several of their constituent variables—such as admission glucose, onset-to-treatment time, and early infarct or hyperdense-artery signs—were not systematically recorded in this retrospective dataset. These comparisons should also be read horizontally. The reference scores were developed largely in North American and European populations and have been externally validated mainly in European and East Asian cohorts, with discrimination varying appreciably by setting [8,22,23,27]. Multi-ethnic registry evidence reinforces this caution: in 8825 South Asian patients managed within a single health system in Qatar, ethnicity was independently associated with poor 90-day functional outcome even after adjustment for admission severity, while baseline NIHSS remained the dominant predictor (adjusted odds ratios 4.86 and 20.37 for moderate and severe strokes) [28]. That report concurs with our central finding regarding the primacy of neurological severity, yet simultaneously demonstrates that outcome distributions and prognostic calibration shift across ethnic groups even under a shared care pathway. Our cohort is single-center and ethnically homogeneous, and is therefore constrained precisely along this dimension; the present model should be regarded as contemporary evidence from an under-represented population rather than as a general-purpose instrument. External validation in cohorts where multiple scores can be computed simultaneously would enable more rigorous comparison and represents an important direction for future work. Machine-learning approaches have likewise been applied to this prediction task and can attain comparable discrimination, while raising additional requirements for interpretability and calibration [42].

### 4.4. Interpretation of the Functional Trajectory

The functional trajectory summarized in Table 5 reflects the interplay of early mortality and subsequent recovery in survivors. Between discharge and 30 days, the proportion of patients with good functional outcome (mRS 0–2) declined from 55.8% to 41.7% despite no additional recorded mortality in that interval. This early decline may reflect post-discharge complications and changes in support intensity following transfer from acute inpatient care to home or community rehabilitation—such as reduced nursing support, medication-adherence difficulties, post-stroke fatigue, and early complications including urinary tract infection or aspiration pneumonia—although these mechanisms were not directly measured in this study. Between 3 months and 12 months, the proportion of patients achieving mRS 0–2 partially recovered to 53.9%, consistent with expected rehabilitation-related improvement among survivors. This pattern accords with the recovery profiles described in contemporary stroke rehabilitation guidance and with the established biological time course of post-stroke motor recovery [43,44]. Fourteen additional deaths were recorded between 3 months and 1 year, yielding a cumulative 1-year mortality of 44.2%.

### 4.5. Clinical Implications

The clinical value of the present analysis lies in the demonstration that a three-variable model using NIHSS, ASPECTS, and age—all obtainable within minutes of emergency department arrival—achieves discrimination comparable to more elaborate composite scores in this cohort. For treating clinicians, this supports rapid bedside risk stratification at admission without waiting for laboratory results, occlusion characterization, or reperfusion status. Given that validation was internal and calibration was imperfect in the intermediate-risk range, these estimates should at present support research-oriented risk stratification and hypothesis generation rather than direct individual treatment decisions; for rehabilitation services, admission-based risk tiers may help anticipate resource needs pending external validation. The provided prediction equation and risk table (Supplementary Table S7) are intended to facilitate the transparent application at the point of care rather than to replace clinical judgment.

### 4.6. Limitations

Several limitations warrant explicit acknowledgment. First, the retrospective single-center design and the concentrated case-mix severity limit the direct generalizability of the AUC estimates; multicenter external validation is required before clinical implementation is considered. Because standard ASPECTS was developed for the middle cerebral artery territory, the cohort was restricted to anterior-circulation strokes, and the model does not apply to posterior-circulation events. In addition, because outcome data were abstracted retrospectively from routine records, it could not be confirmed that outcome assessors were blinded to baseline predictor values. Second, although the parsimonious specification was prespecified on the basis of admission availability, several potentially prognostic variables—including 24 h NIHSS change, National Institutes of Health Stroke Scale improvement after reperfusion, and detailed occlusion characterization—were not consistently documented and could not be evaluated. Third, infarct volume was measured on follow-up imaging and is therefore not a true admission predictor; it is presented in baseline tables as a descriptive variable only. Fourth, the 3-month primary endpoint reflects the widely used benchmark for outcome studies but does not capture continued recovery beyond one year, which may follow different trajectories in specific subgroups. Fifth, the THRIVE score could be calculated in this cohort and was compared directly with the prespecified model (Section 3.12); however, ASTRAL, DRAGON, and iScore could not be evaluated head-to-head because several of their required components—including admission glucose, onset-to-treatment time, and specific early imaging signs—were not systematically retrievable from the retrospective records. Sixth, calibration was imperfect. Although the internally validated calibration slope (0.958) and calibration-in-the-large (0.009) indicate close agreement between predicted and observed risk overall, the Hosmer–Lemeshow test was statistically significant (χ^2^ = 25.8, df = 8, *p* = 0.001), and the flexible calibration curve (Supplementary Figure S1) localizes this deviation to the intermediate-risk range, where the model over-predicted observed risk in the third decile and under-predicted it in the fourth. Predictions in this intermediate band should therefore be treated with corresponding caution, whereas calibration in the low- and high-risk ranges—where most clinical decisions in this cohort would fall—was close to ideal. Because the Hosmer–Lemeshow test is sensitive to bin count and sample size, the flexible calibration curve rather than the test statistic should carry the greater interpretive weight, and external validation remains necessary to establish whether this deviation is reproducible. For the same reason, the nomogram (Figure 4) and the bedside risk table (Supplementary Table S7) should be used to stratify patients into broad risk tiers rather than to quote exact individual probabilities, particularly for patients falling in the intermediate-risk band. Seventh, exact discharge and 30-day mRS values were unavailable for 120 patients who died between 30 days and 3 months, and their mRS band at those two timepoints was reconstructed from discharge destination and subsequent mortality. Although this reconstruction was applied transparently and is reported in full, it relies partly on subsequent outcome information and may have introduced misclassification into the discharge and 30-day distributions; the early functional trajectory before 3 months should therefore be regarded as exploratory. The 3-month primary endpoint, which is based on directly recorded mRS, is unaffected by this reconstruction.

## 5. Conclusions

In this contemporary Turkish tertiary center cohort of 432 adults with anterior-circulation acute ischemic stroke, baseline NIHSS was the dominant admission predictor of 3-month functional outcome, and early ASPECTS did not add measurable independent prognostic value once neurological severity and age were accounted for. A prespecified three-variable admission model achieved excellent internal discrimination and good overall calibration, albeit with localized miscalibration in the intermediate-risk range, matched the performance of a more comprehensive ten-variable specification, and remained robust under age-modeling and bootstrap sensitivity analyses. It is important to distinguish the two time horizons examined here: the model was developed and internally validated for the 3-month endpoint only, whereas the one-year data describe the observed functional trajectory of this cohort rather than a separate, validated 12-month prediction model. Among survivors, functional status continued to improve between 3 months and 1 year, while cumulative mortality rose from 41.0% to 44.2%; an early prediction of poor 3-month outcome should therefore not be read as a fixed long-term prognosis, and prediction of 12-month recovery requires dedicated modeling in adequately powered cohorts. An exploratory 12-month model is reported in Section 3.13 and demonstrates the point directly: because 96.0% of poor 12-month outcomes in this cohort were deaths, such a model reduces in substance to a mortality model and cannot inform functional recovery among survivors. These findings provide preliminary evidence that admission-based clinical severity may be sufficient for early 3-month risk stratification in ischemic stroke populations comparable to ours. Because validation was internal and confined to a single regional tertiary center with a distinctive case-mix, reperfusion rate, and age distribution, the model must not be assumed to apply universally to all stroke populations. External validation in independent multicenter cohorts with different case-mix profiles is required before the model can be recommended for clinical implementation, and prospective evaluation of whether model-guided prognostication improves resource allocation, family communication, or patient-centered outcomes remains an important priority for future research.

## Figures and Tables

**Table 1:** Baseline characteristics by 3-month functional outcome.

Variable	Good Outcome (n = 180)	Poor Outcome (n = 252)
Age, years, mean ± SD	62.7 ± 12.1	66.9 ± 13.2 †
Age ≥ 65 years, n (%)	81 (45.0)	142 (56.3) †
Male, n (%)	101 (56.1)	144 (57.1)
NIHSS, mean ± SD	4.6 ± 4.1	18.4 ± 10.9 ‡
GCS, mean ± SD	13.2 ± 1.8	9.8 ± 3.4 ‡
ASPECTS, mean ± SD	9.0 ± 1.0	6.0 ± 2.8 ‡
Infarct volume, mL, mean ± SD	28.7 ± 23.6	98.7 ± 80.4 ‡
Hypertension, n (%)	125 (69.4)	185 (73.4)
Diabetes mellitus, n (%)	62 (34.4)	95 (37.7)
Atrial fibrillation, n (%)	59 (32.8)	105 (41.7)
Previous stroke, n (%)	35 (19.4)	55 (21.8)
IV thrombolysis, n (%)	18 (10.0)	50 (19.8) †
Mechanical thrombectomy, n (%)	9 (5.0)	44 (17.5) ‡

† *p* < 0.05; ‡ *p* < 0.001 versus the good-outcome group. Infarct volume was measured on follow-up imaging at 24–72 h using the ABC/2 method and is presented as a descriptive variable only; it was not included in the primary admission-based model. *p*-values compare the good- and poor-outcome groups. NIHSS, National Institutes of Health Stroke Scale; GCS, Glasgow Coma Scale; ASPECTS, Alberta Stroke Program Early CT Score; IV, intravenous; SD, standard deviation.

**Table 2:** Functional outcomes by baseline NIHSS category.

NIHSS Category	n (%)	Good Outcome mRS 0–2	Poor Outcome mRS 3–6	3-Month Mortality
Minor (0–4)	130 (30.1)	118 (90.8%)	12 (9.2%)	8 (6.2%)
Moderate (5–15)	167 (38.7)	62 (37.1%)	105 (62.9%)	61 (36.5%)
Moderate-Severe (16–24)	65 (15.0)	0 (0.0%)	65 (100.0%)	49 (75.4%)
Severe (>24)	70 (16.2)	0 (0.0%)	70 (100.0%)	59 (84.3%)

NIHSS, National Institutes of Health Stroke Scale; mRS, modified Rankin Scale.

**Table 3:** Functional outcomes by baseline ASPECTS category.

ASPECTS Category	n (%)	Good Outcome mRS 0–2	Poor Outcome mRS 3–6	3-Month Mortality
≤5 (Severe ischemia)	86 (19.9)	0 (0.0%)	86 (100.0%)	70 (81.4%)
6–7 (Moderate)	96 (22.2)	18 (18.8%)	78 (81.2%)	57 (59.4%)
8–10 (Mild)	250 (57.9)	162 (64.8%)	88 (35.2%)	50 (20.0%)

ASPECTS, Alberta Stroke Program Early CT Score; mRS, modified Rankin Scale.

**Table 4:** Multivariate logistic regression analysis for poor functional outcome (mRS 3–6) at 3 months.

Variable	Odds Ratio	95% CI	*p*-Value
NIHSS (per 5-point increase)	4.16	2.79–6.22	<0.001
ASPECTS (per 1-point increase)	0.99	0.73–1.34	0.952
Age ≥ 65 years	0.79	0.46–1.37	0.405

NIHSS, National Institutes of Health Stroke Scale; ASPECTS, Alberta Stroke Program Early CT Score; CI, confidence interval; AUC, area under the receiver operating characteristic curve; mRS, modified Rankin Scale.

**Table 5:** Functional trajectory over time.

Timepoint	mRS 0–2, n (%)	mRS 3–5, n (%)	mRS 6 (Death), n (%)
Discharge	241 (55.8)	134 (31.0)	57 (13.2)
30 days	180 (41.7)	195 (45.1)	57 (13.2)
3 months	180 (41.7)	75 (17.4)	177 (41.0)
6 months	212 (49.1)	43 (10.0)	177 (41.0)
1 year	233 (53.9)	8 (1.9)	191 (44.2)

Mean mRS is not reported because mRS 6 denotes death. In-hospital mortality (57; 13.2%) was derived from the recorded discharge destination and agreed with the 30-day mortality field in all 57 cases. For 120 patients who were discharged alive but died between 30 days and 3 months, exact discharge and 30-day mRS values could not be retrieved; these patients were assigned to the mRS 3–5 band at those timepoints using a post hoc, rule-based reconstruction based on discharge destination (64 nursing facility, 34 palliative care, 22 home) and subsequent mortality. This reconstruction affects only the discharge and 30-day rows; the 3-month primary endpoint is unaffected. The 6-month mRS was available for all 432 patients and indicated that the 14 patients who died between 3 months and 1 year were alive at 6 months. Exact dates of death after the 6-month assessment were unavailable. mRS, modified Rankin Scale.

## Data Availability

The datasets generated and analyzed during the current study are not publicly available due to institutional policy and patient privacy considerations but are available from the corresponding author on reasonable request and subject to institutional data-sharing procedures.

## Supplementary Materials

The following supporting information can be downloaded at https://www.mdpi.com/article/10.3390/jcm15156016/s1, Figure S1: Calibration plot for the primary prediction model. Table S1: STROBE Statement Checklist. Table S2: DeLong test results for nested model comparison. Table S3: Net reclassification improvement (NRI) and integrated discrimination improvement (IDI) analyses. Table S4: Variance inflation factors and Spearman correlations among predictors. Table S5: Comprehensive multivariable model with ten predictors. Table S6: Age modeling sensitivity analysis. Table S7: Bedside risk table with prediction equation. Table S8: TRIPOD reporting checklist for prediction model development. Table S9: Exploratory 12-month outcome model. Table S10: Head-to-head comparison with the THRIVE score.
