# Supplementary material for: Baseline Neurological Severity and Early Imaging Findings Predict 3-Month Functional Outcome in Anterior-Circulation Acute Ischemic Stroke: A Retrospective Cohort Study with One-Year Follow-Up
Source: J Clin Med. 2026 Aug 2;15(15):6016. doi: 10.3390/jcm15156016 (PMC13467213; doi:10.3390/jcm15156016)
Supplement: Supplementary file 1 [file jcm-15-06016-s001.zip › jcm-4431511-supplementary.pdf]

# Supplementary Materials

## Supplementary Materials

**Baseline Neurological Severity and Early Imaging Findings Predict 3-Month Functional Outcome in Anterior-Circulation Acute Ischemic Stroke: A Retrospective Cohort Study with One-Year Follow-Up**

Manuscript ID: jcm-4431511

All analyses were performed on the full analytic cohort (n = 432). All statistics reported below were computed directly from the source dataset.

### Table S1. STROBE checklist for cohort studies

| No. | Item | Reported in |
|---|---|---|
| 1a | Study design indicated in title/abstract | Title; Abstract |
| 1b | Informative and balanced abstract | Abstract |
| 2 | Scientific background and rationale | Section 1 |
| 3 | Objectives and prespecified hypotheses | Section 1 (final paragraph) |
| 4 | Study design presented early | Section 2.1 |
| 5 | Setting, locations, recruitment/follow-up dates | Section 2.1 |
| 6a | Eligibility criteria, sources, selection methods | Section 2.2 |
| 6b | Matching criteria (not applicable) | Not applicable |
| 7 | Outcomes, exposures, predictors, confounders defined | Sections 2.3–2.4 |
| 8 | Sources of data and measurement methods | Sections 2.2–2.3 |
| 9 | Efforts to address potential bias | Sections 2.4; 4.6 |
| 10 | Study size explanation | Section 2.6 (events-per-variable) |
| 11 | Handling of quantitative variables and groupings | Sections 2.3–2.4; Table S6 |
| 12a | Statistical methods, confounding control | Section 2.5 |
| 12b | Subgroup and interaction methods | Section 2.5; Tables S5–S6 |
| 12c | Handling of missing data | Section 2.3 (no missing primary data); Table 5 footnote (longitudinal) |
| 12d | Loss to follow-up addressed | Section 3.8; Table 5 footnote |

| No. | Item | Reported in |
|---|---|---|
| 12e | Sensitivity analyses | Sections 3.10–3.11; Tables S5–S6 |
| 13 | Numbers of participants at each stage | Section 3.1; Figure 1 |
| 14a | Characteristics of participants | Table 1 |
| 14b | Number with missing data per variable | Section 3.1; Table 5 footnote |
| 14c | Follow-up time summarised | Section 3.8 |
| 15 | Numbers of outcome events | Section 3.3; Tables 2–3 |
| 16a | Unadjusted and adjusted estimates with CI | Tables 2–4; Table S5 |
| 16b | Category boundaries when continuous variables categorised | Tables 2–3; Section 2.3 |
| 17 | Other analyses performed | Sections 3.9–3.13; Tables S2–S6 and S9–S10 |
| 18 | Key results with reference to objectives | Section 4 (opening) |
| 19 | Limitations | Section 4.6 |
| 20 | Cautious overall interpretation | Sections 4.1; 4.7 |
| 21 | Generalisability | Section 4.6 |
| 22 | Funding | Funding statement |

## Table S2. DeLong test for the incremental discriminative value of ASPECTS

| Model | Predictors | AUC | 95% CI |
|---|---|---|---|
| Reduced | NIHSS (per 5 points) + age ≥65 years | 0.9031 | 0.876–0.931 |
| Full | NIHSS (per 5 points) + ASPECTS + age ≥65 years | 0.9035 | 0.876–0.931 |

| Comparison | ΔAUC | DeLong z | p-value |
|---|---|---|---|
| Full vs reduced | +0.0003 | 0.679 | 0.497 |

### Likelihood ratio test (same nested models)

| Model | Log-likelihood | LR $\chi^2$ | df | p-value |
|---|---|---|---|---|
| Reduced (NIHSS + age) | −166.821 | — | — | — |
| Full (+ ASPECTS) | −166.819 | 0.004 | 1 | 0.952 |

Adding ASPECTS to a model already containing NIHSS and age produced no statistically or clinically meaningful gain in discrimination, and did not improve model fit. DeLong's test for two correlated ROC curves was used for the AUC comparison; the likelihood ratio test compares the fit of the nested models directly. The two tests, together with the reclassification metrics in Table S3 and the adjusted odds ratio for ASPECTS in Table 4 (p = 0.952), are mutually consistent. AUC, area under the receiver operating characteristic curve; CI, confidence interval.

## Table S3. Net reclassification improvement and integrated discrimination improvement

Reference model: NIHSS + age ≥65 years. Comparison model: NIHSS + ASPECTS + age ≥65 years.

| Metric | Estimate | 95% CI |
|---|---|---|
| Category-free NRI (events) | +0.103 | −0.02 to 0.23 |
| Category-free NRI (non-events) | +0.122 | −0.01 to 0.25 |
| Category-free NRI (overall) | +0.225 | 0.04–0.44 |
| IDI | −0.0001 | −0.003 to 0.003 |

The overall category-free NRI was driven by small, offsetting movements in predicted probability rather than by reclassification across clinically meaningful risk strata. The IDI was essentially zero, indicating no measurable improvement in the separation of predicted probabilities between events and non-events. Taken together with the DeLong result (Table S2), these findings indicate that ASPECTS did not improve the AUC, the likelihood-based model fit, or the IDI, despite a positive category-free NRI whose clinical significance is uncertain once baseline NIHSS is in the model. NRI, net reclassification improvement; IDI, integrated discrimination improvement.

## Table S4. Multicollinearity assessment

**Panel A. Spearman rank correlations**

| Variable pair | ρ | p-value |
|---|---|---|
| NIHSS – ASPECTS | −0.886 | <0.001 |
| NIHSS – GCS | −0.780 | <0.001 |
| ASPECTS – infarct volume | −0.837 | <0.001 |

**Panel B. Variance inflation factors (primary model)**

| Variable | VIF |
|---|---|
| NIHSS (per 5-point increase) | 6.02 |
| ASPECTS (per 1-point increase) | 5.98 |
| Age ≥65 years | 1.06 |

Variance inflation factors were computed from the design matrix including the intercept. Both NIHSS and ASPECTS exceed the conventional threshold of 5, and their rank correlation exceeds the conventional threshold of 0.7, indicating moderate-to-substantial shared variance between the clinical and imaging measures of stroke extent. This shared variance, rather than an absence of prognostic content, accounts for the wide confidence interval and non-significance of ASPECTS in the multivariable model (Table 4). VIF, variance inflation factor; GCS, Glasgow Coma Scale.

## Table S5. Comprehensive multivariable model

| Variable | Adjusted OR | 95% CI | p-value |
|---|---|---|---|
| NIHSS (per 5-point increase) | 4.16 | 2.63–6.58 | <0.001 |
| ASPECTS (per 1-point increase) | 0.94 | 0.66–1.33 | 0.719 |
| Age ≥65 years | 0.82 | 0.46–1.45 | 0.491 |
| Infarct volume (per mL) | 0.99 | 0.98–1.01 | 0.442 |
| GCS (per 1-point increase) | 1.02 | 0.87–1.20 | 0.820 |
| Hypertension | 0.93 | 0.51–1.69 | 0.813 |
| Diabetes mellitus | 1.01 | 0.56–1.82 | 0.986 |
| Atrial fibrillation | 1.16 | 0.65–2.08 | 0.615 |
| IV thrombolysis | 1.86 | 0.93–3.72 | 0.082 |
| Mechanical thrombectomy | 1.51 | 0.62–3.69 | 0.363 |

Intercept = −1.896.

**Model comparison**

| Model | Predictors | AUC | AIC | CV AUC | CV calibration slope | CV calibration-in-the-large | CV Brier score |
|---|---|---|---|---|---|---|---|
| Parsimonious | 3 | 0.904 | 341.6 | 0.899 | 0.958 | 0.009 | 0.130 |
| Comprehensive | 10 | 0.905 | 350.8 | 0.896 ± 0.017 | 0.887 | 0.000 | 0.134 |
| NIHSS alone | 1 | 0.903 | 338.3 | — | — | — | — |

The two models were compared on discrimination (AUC, cross-validated AUC), model fit (AIC), and calibration (5-fold cross-validated calibration slope, calibration-in-the-large, and Brier score). The comprehensive model achieved essentially identical discrimination (ΔAUC +0.0016) at the cost of a higher AIC, indicating inferior fit once model complexity is penalised, and its cross-validated calibration was slightly worse than that of the parsimonious model (calibration slope 0.887 versus 0.958; Brier score 0.134 versus 0.130). Baseline NIHSS remained the only statistically significant predictor. For reference, a model containing NIHSS alone achieved the lowest AIC of the three; the three-predictor model is retained as the primary model because the study question concerns the incremental value of ASPECTS and age over NIHSS, which requires those terms to be present in the model. AIC, Akaike information criterion; CV, 5-fold cross-validated. Calibration slope and calibration-in-the-large were obtained by regressing observed outcomes on the cross-validated linear predictor; a slope below 1 indicates mild overfitting of predicted risks.

## Table S6. Age modelling sensitivity analysis

| Age specification | AUC | AIC | Age effect estimate | p-value |
|---|---|---|---|---|
| Dichotomous (≥65 years) | 0.9035 | 341.6 | OR 0.79 (95% CI 0.46–1.37) | 0.405 |

| Age specification | AUC | AIC | Age effect estimate | p-value |
|---|---|---|---|---|
| Continuous (per year) | 0.9033 | 341.5 | OR 0.99 (95% CI 0.97–1.01) | 0.369 |
| Polynomial (age + age$^2$) | 0.9032 | 343.5 | — | — |

All three specifications were fitted alongside NIHSS (per 5-point increase) and ASPECTS. Discrimination was essentially identical across specifications and the AIC differences were negligible (dichotomous versus continuous, ΔAIC 0.1). Age was not independently associated with 3-month outcome under any specification. Dichotomisation at 65 years therefore did not distort the estimated effects, and the dichotomous form was retained for clinical interpretability.

## Table S7. Bedside risk table (approximate predicted probability) and prediction equation

**Prediction equation**

$$\text{logit}(P) = -2.116 + 1.426 \times (\text{NIHSS} / 5) - 0.0094 \times \text{ASPECTS} - 0.232 \times I(\text{Age} \geq 65 \text{ years})$$

P(poor outcome at 3 months) = 1 / (1 + e^(−logit(P)))

**Predicted probability of poor functional outcome (mRS 3–6) at 3 months**

| NIHSS category (value used) | ASPECTS 8–10 (9), age <65 | ASPECTS 8–10 (9), age ≥65 | ASPECTS 6–7 (7), age <65 | ASPECTS 6–7 (7), age ≥65 | ASPECTS ≤5 (4), age <65 | ASPECTS ≤5 (4), age ≥65 |
|---|---|---|---|---|---|---|
| Minor, 0–4 (2) | 16% | 14% | 17% | 14% | 17% | 14% |
| Moderate, 5–15 (10) | 66% | 60% | 66% | 61% | 67% | 62% |
| Moderate–severe, 16–24 (20) | 97% | 96% | 97% | 96% | 97% | 97% |
| Severe, >24 (28) | >99% | >99% | >99% | >99% | >99% | >99% |

**This table is intended as an aid to risk stratification — that is, to ranking patients from lower to higher risk — rather than as a source of exact individual probability estimates.** Calibration was close to ideal in the low- and high-risk ranges but deviated in the intermediate-risk range (Figure S1); values in the middle of the table therefore carry greater uncertainty than their apparent precision suggests, and should be read as indicating a risk tier rather than a point estimate. Probabilities are computed from the equation above using the representative value shown in parentheses for each category. The Youden-derived decision threshold was 0.32 (sensitivity 90.1%, specificity 71.1%, positive predictive value 81.4%, negative predictive value 83.7%). Reading across any row demonstrates that ASPECTS and age alter the predicted probability by only a few

percentage points, whereas reading down any column demonstrates the dominant contribution of baseline NIHSS. mRS, modified Rankin Scale.

## Table S8. TRIPOD checklist (development of a prediction model)

| No. | Item | Reported in |
|---|---|---|
| 1 | Title identifies study as developing a prediction model | Title |
| 2 | Abstract: objectives, setting, participants, outcome, predictors, statistics, results, conclusions | Abstract |
| 3a | Background and rationale, reference to existing models | Section 1 |
| 3b | Objectives, including development or validation | Section 1 (final paragraph) |
| 4a | Source of data (cohort design) | Section 2.1 |
| 4b | Key study dates | Section 2.1 |
| 5a | Study setting, locations, relevant dates | Section 2.1 |
| 5b | Eligibility criteria | Section 2.2 |
| 5c | Treatments received | Section 2.2; Table 1 |
| 6a | Outcome definition and assessment | Section 2.4 |
| 6b | Blinding of outcome assessment | Section 2.4; blinding of outcome assessors could not be verified because outcomes were abstracted retrospectively (Section 4.6) |
| 7a | Predictor definition and measurement, blinding of assessors | Section 2.3 (ASPECTS assessed by three stroke neurologists blinded to outcome) |
| 7b | Blinding of predictor assessment | Section 2.3 |
| 8 | Sample size rationale | Section 2.6 |
| 9 | Handling of missing data | Sections 2.3–2.4; Table 5 footnote |
| 10a | Handling of predictors in the analysis | Section 2.5; Table S6 |
| 10b | Model type, predictor selection, internal validation | Section 2.5 |
| 10c | Internal validation methods | Section 2.5 (5-fold cross-validation; bootstrap optimism correction) |
| 10d | Measures of model performance | Section 2.5; Section 3.7 |
| 11 | Risk groups created | Tables 2–3; Table S7 |
| 13a | Participant flow, follow-up, numbers | Section 3.1; Figure 1 |

| No. | Item | Reported in |
|---|---|---|
| 13b | Participant characteristics, missing data | Table 1; Section 3.1 |
| 14a | Number of participants and outcome events | Section 3.3 |
| 14b | Unadjusted association of predictors with outcome | Tables 2–3 |
| 15a | Full prediction model (all regression coefficients and intercept) | Section 3.7; Table S7 |
| 15b | Explanation of how to use the model | Figure 4; Table S7 |
| 16 | Model performance measures with CI | Section 3.7; Figure S1; Tables S2–S3 |
| 17 | Model updating (not applicable) | Not applicable |
| 18 | Limitations | Section 4.6 |
| 19b | Interpretation of results | Sections 4.1–4.4 |
| 20 | Potential clinical use and implications | Sections 4.5; 4.7 |
| 21 | Supplementary resources | This document |
| 22 | Funding | Funding statement |

Item 10c (internal validation methods) applies to this development study and is addressed in Section 2.5. Items 10e, 12, 13c and 19a apply only to external validation studies and are not applicable.

## Figure S1. Calibration of the prediction model

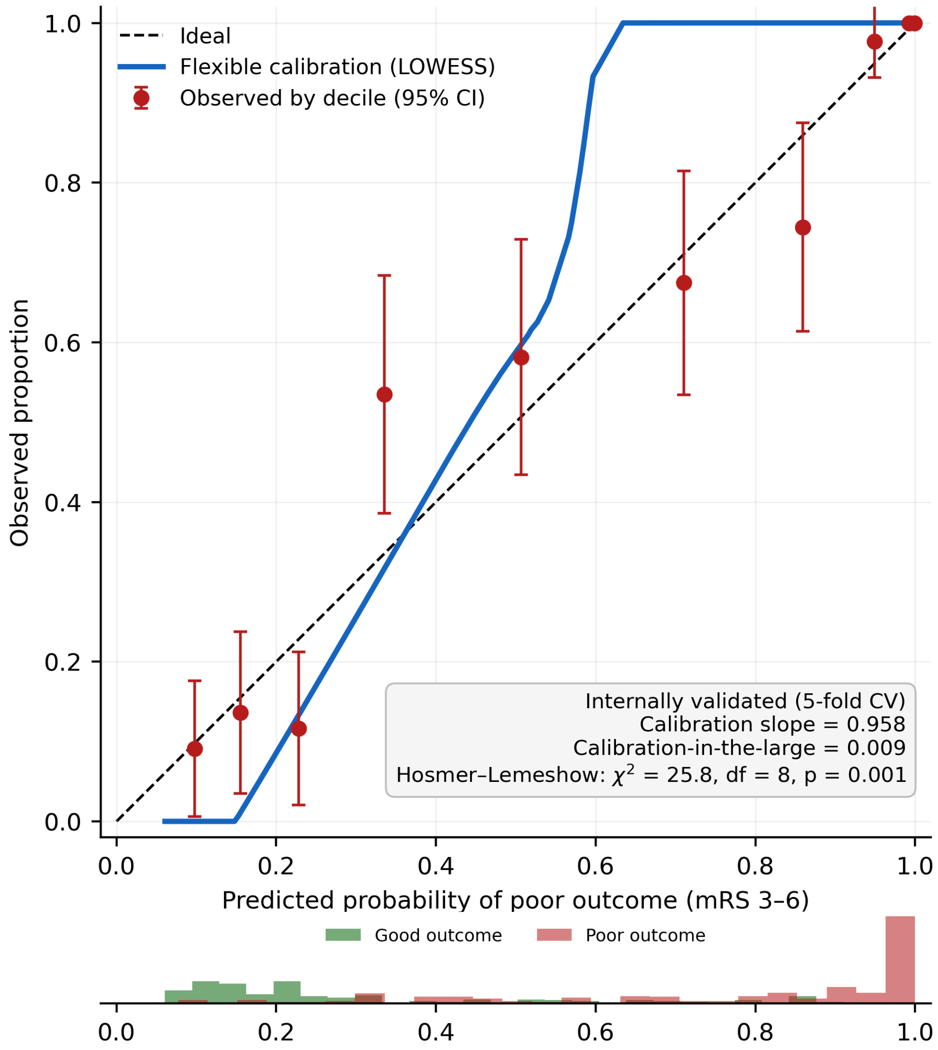


**Figure S1.** Flexible calibration plot for the model containing baseline NIHSS (per 5-point increase), ASPECTS, and age ≥65 years, predicting poor functional outcome (mRS 3–6) at 3 months. Predicted probabilities were obtained by 5-fold stratified cross-validation, so the plot reflects internally validated rather than apparent calibration; apparent calibration statistics computed in the derivation sample are uninformative by construction (slope exactly 1.00, calibration-in-the-large exactly 0.00). The dashed diagonal represents ideal calibration. The solid curve is a LOWESS smoother through the cross-validated predictions. Red points show the observed event proportion within each decile of predicted risk, with 95% confidence intervals. The internally validated calibration slope was 0.958 and calibration-in-the-large was 0.009, close to their target values; however, the flexible curve and the Hosmer–Lemeshow test demonstrated clinically relevant local miscalibration in the intermediate-risk range. The Hosmer–Lemeshow test was statistically significant ($\chi^2$ = 25.8, df = 8, p = 0.001): the model over-predicted observed risk in the third decile of predicted risk (5 events observed versus 9.8 expected) and under-predicted it in the fourth (23 observed versus 14.4 expected), while calibration in the low- and high-risk ranges was

close to ideal. This localised miscalibration in the intermediate-risk range is discussed in Sections 3.7 and 4.6. The histogram below the plot shows the distribution of cross-validated predicted probabilities among patients with good and poor outcome.

## Table S9. Exploratory 12-month outcome model

Same three admission predictors as the primary model, fitted to poor functional outcome (mRS 3–6) at 12 months. Reported as exploratory; see Section 3.13.

**Panel A. Model coefficients**

| Variable | Adjusted OR | 95% CI | p-value |
|---|---|---|---|
| NIHSS (per 5-point increase) | 2.97 | 2.16–4.09 | <0.001 |
| ASPECTS (per 1-point increase) | 1.18 | 0.94–1.49 | 0.142 |
| Age ≥65 years | 0.63 | 0.38–1.05 | 0.076 |

Intercept = −3.760.

**Panel B. Comparison across time horizons**

| Endpoint | Events | Non-events | AUC | AIC | NIHSS aOR (95% CI) |
|---|---|---|---|---|---|
| Poor outcome (mRS 3–6) at 3 months | 252 | 180 | 0.904 | 341.6 | 4.16 (2.79–6.22) |
| Poor outcome (mRS 3–6) at 12 months | 199 | 233 | 0.868 | 409.7 | 2.97 (2.16–4.09) |
| All-cause mortality at 12 months | 191 | 241 | 0.857 | 420.5 | 2.80 (2.06–3.80) |

## Table S10. Head-to-head comparison with the THRIVE score

Discrimination of the THRIVE score and the prespecified NIHSS–ASPECTS–age model for 3-month poor functional outcome (mRS 3–6) in the same 432-patient cohort. AUCs were compared using DeLong's test for correlated ROC curves.

| Model | AUC | 95% CI | DeLong comparison |
|---|---|---|---|
| THRIVE | 0.792 | 0.751–0.834 | Reference |
| NIHSS + ASPECTS + age | 0.904 | 0.876–0.932 | p < 0.001 |

THRIVE was calculated according to its original definition: age (≤59 = 0, 60–79 = 1, ≥80 = 2 points), baseline NIHSS (≤10 = 0, 11–20 = 2, ≥21 = 4 points), and one point each for a history of hypertension, diabetes mellitus, and atrial fibrillation (total range 0–9). AUC, area under the receiver operating characteristic curve; CI, confidence interval; NIHSS, National Institutes of Health Stroke Scale; ASPECTS, Alberta Stroke Program Early CT Score.

**Panel C. Why the 12-month model reduces to a mortality model**

| Diagnostic | Value |
| --- | --- |
| Poor 12-month outcomes that were deaths | 191 / 199 (96.0%) |
| Surviving patients in the mRS 3–5 stratum at 12 months | 8 |
| Correlation between predicted probabilities from the 12-month poor-outcome model and the 12-month mortality model | Pearson r = 0.999 |
| Discrimination of surviving dependent patients (mRS 3–5) from decedents (mRS 6) within the poor-outcome group | AUC 0.563 |

The 12-month poor-outcome model is estimable and discriminates adequately, but its predictions are almost perfectly collinear with those of a pure 12-month mortality model, and it cannot separate the 8 surviving dependent patients from the 191 decedents. The composite endpoint at 12 months is therefore dominated by death, and a model fitted to it does not inform functional recovery among survivors. Establishing a validated 12-month functional recovery model would require a cohort containing substantially more surviving dependent patients. Confidence intervals are Wald-based. AUC, area under the receiver operating characteristic curve; AIC, Akaike information criterion; mRS, modified Rankin Scale.
